# Some Recent Novelties in Diagnostic Technique

**Published:** 1914-03-28

**Authors:** 


### SOME RECENT NOVELTIES IN DIAGNOSTIC TECHNIQUE.

The post-graduate paper, *Zeitschrift fuer Aerztliche Fortbildung*, gives, in every number, a section devoted to the description of modern methods of diagnostic technique. Some of these diagnostic hints are contributed by general practitioners and are not to be found even in modern text-books. We have, therefore, thought it desirable to give a summary of some of the notes contained in the last two numbers in the hope that it may be of use to our readers.

### Fluorescein as an Indicator of Kidney Integrity.

Professor Strauss has lately been experimenting with the sodium salt of fluorescein, which is put on the market under the trade name of "Uranin." He has found this an admirable indicator of the functional integrity of the kidneys, and his method of applying the test is exceedingly simple. The patient is given fifteen grains of the salt (1 gramme) dissolved in tea, coffee, or cocoa, and taken on an empty stomach. In a patient whose kidneys are functioning normally there is marked fluorescence of the urine passed from ten to twenty minutes after the exhibition of the drug, which lasts for thirty hours. Slight traces of fluorescence are accentuated when the urine is mixed with ammonia. If the fluorescence lasts longer than forty hours it is an indication of the fact that the kidneys are not functioning properly; fluorescence of the urine fifty hours after exhibition of the drug points to a gross kidney lesion. Strauss thinks that the soda salt is the best indicator we possess, since it is not vitiated by individual idiosyncrasy on the part of many patients as are some other indicators.

### Method of Demonstrating Occult Fat in Fæces.

Dr. Saathoff, of Obersdorf, advises the following technique for demonstrating and estimating occult fat in fæces. A small portion of fæcal substance is roughly smeared on the slide and slightly condensed over the flame of a Bunsen or spirit lamp. One or two drops of a mixture of glacial acetic acid (90 c.cm.) and absolute alcohol (10 c.cm.), in which some Sudan II. has been dissolved, are then dropped on the preparation with which it is intimately mixed with the end of a wooden match. When this is carefully done the preparation has the appearance of a fairly thick gruel of a vivid red colour. A thin cover slip is now laid over the preparation, and the slide is warmed and immediately examined under the microscope. The fat shows itself in the form of yellow or red drops which are easily seen, even to the smallest particles, so that it is comparatively easy to make a rough estimate of the amount present in comparison with the rest of the fæcal material. When the slide cools the fat crystallises in needle-shaped crystals of fatty acids which disappear on warming. A normal adult evacuates daily approximately 6 gramme (90 grains) of fat in the fæces, and normal dry fæces contains 20 per cent. of fat. Saathoff has found his method particularly useful in showing an increased evacuation of fat in cases of thyroid disease, hepatic lesions, pancreatic disorders, and diseases of the bowel.

### To Demonstrate the Presence of Iodine in Urine.

Dr. Ehrmann, discussing the various methods available for detecting the presence of iodine in urine, advises the following method: Some 2 c.cm. of the suspected urine is mixed in a test tube with 1 c.cm. of dilute nitric acid and 0.5 c.cm. of hydrogen peroxide solution. The mixture is intimately mixed, allowed to stand for a few minutes, and then shaken up with 10 c.cm. of chloroform or toluol. The presence of iodine in the urine is indicated by the chloroform or toluol becoming reddened. The test answers well with minute quantities of iodine, and is an exceedingly useful one to remember.

### A New Method for the Differential Blood Count.

Dr. Dunzelt describes his method as follows: Two standard solutions are prepared according to the following recipe:

| | | |
|---|---|---|
| A. | Methyl blue ... ... ... | 0.08 gramme. |
| | Aq. dest. ad ... ... ... | 50.00 gramme. |
| | | Filtra. |
| B. | 1 per cent. aqueous sol. eosin... | 5.0 gramme. |
| | Aceton pur. ... ... ... | 30.0 gramme. |
| | Aq. dest. ad ... ... ... | 100.0 gramme. |
| | | Filtra. |

20 c.cm. of A is mixed with 40 c.cm. of B; the filtered solution of the mixture is the working stain, which can only be used for a few days, and is best freshly prepared. When a blood examination has to be made quickly A and B may be mixed in the proportion of 1 to 2. The technique of the examination is as with ordinary methods. The blood is mixed in the pipette with the staining solution and then placed in the slide chamber. The red corpuscles are not visible, the solution is quite transparent, and even the deformed leucocytes are clearly demonstrated, the definitions being clear and sharp. In cases of eosinophilia and lymphocytosis the new method is said to be a great improvement upon the older methods.

